# The Effects of Adolescents’ Participation in Video Games on Cognitive Function and Motor Control Skills

**DOI:** 10.3390/healthcare11202740

**Published:** 2023-10-15

**Authors:** Hyoyeon Ahn, Jongeun Won

**Affiliations:** Department of Physical Education, College of Education, Seoul National University, Seoul 08826, Republic of Korea; ahy0522@snu.ac.kr

**Keywords:** video game participation, adolescents, cognitive function, motor control skills

## Abstract

There is still a lack of understanding of the productive areas of video game participation. Therefore, in order to observe positive effects and changes in game participation, this study examines the effects of game participation on the cognitive function and motor skills from 130 adolescents. To evaluate the participants’ test performance, a cognitive function and motor control skill test program consisting of 10 tests were used (Trail Making, Corsi block, Tower of London, shooting game tasks, etc.). Differences in test performances between groups were verified using one-way ANOVA and an independent *t*-test. The results were as follows: first, there was no difference between groups according to the frequency of game participation in every test performance. Second, the results of this study showed statistically significant differences between groups according to the play time of game participation in several test performances (Key-mapping: *p* = 0.40; score of Grid Shot: *p* = 0.01, 0.07; score of Sphere Track: *p* = 0.12, 0.21; accuracy of Sphere Track: *p* = 0.12, 0.16). Also, all the effects’ size results were confirmed as medium (Cohen’s *f* > 0.20 and <0.40). Third, high school students only showed statistically significant higher results in a Multi-tasking test performance than middle school students, and the results of the effect size were confirmed to be middle and large (Multi-tasking1: *p* = 0.00, Cohen’s *d* = 0.830; Multi-tasking2: *p* = 0.05, Cohen’s *d* = 0.501). The results of this study imply the need to regard game participation as a behavior that can contribute to adolescents’ cognitive psychological health.

## 1. Introduction

‘Is game participation truly hazardous?’ In recent years, the debate over the potential risks associated with game participation has garnered international controversy, particularly following the World Health Organization’s (WHO) classification of gaming as a factor contributing to disease and addiction in its 2018 International Classification of Diseases (ICD). Specifically, as reported in the ‘ICD-11 for Mortality and Morbidity Statistics’, the WHO [1] identified adolescents and young adult males aged 12 to 20 years as the most vulnerable group for ‘Gaming Disorder’. The report also linked it to external symptoms such as antisocial behavior and anger control, as well as internal problems like emotional distress and low self-esteem. This classification has raised concerns about excessive worry regarding game participation, as it suggests potential risks such as poor academic performance, school failure, and psychological issues. However, in contrast to this perspective, the WHO encouraged international game participation campaigns under the ‘Play Apart Together’ initiative during the COVID-19 pandemic in 2020 [2]. This clearly acknowledges the positive benefits of participating in games during the pandemic, and international controversy continues due to the WHO’s dual position on game participation.

While game participation is described as risky, there have been studies that examined the impact of gaming stimuli on the human body, particularly focusing on dopamine. Research by Koepp et al. [3] revealed that ‘video game involvement’ leads to a 30–50% increase in dopaminergic neurotransmission levels compared to control task (black screen). Dopamine plays a role in the pleasure-seeking process and can explain the intensity of stimulation. Everyday activities such as enjoying tasty food, engaging in satisfying social interactions, or shopping also activate dopamine. For example, studies have reported that chocolate increases dopamine levels by 55% [4], and nicotine increases them by 150% [5]. Lee et al. [6] argued that compared to substances like drugs, which can increase dopamine levels by up to 1200%, it is difficult to categorize the level of risk associated with increased dopamine levels through game participation. Hence, labeling game participation as inherently hazardous may not be entirely justified due to varying levels of risk compared to substance addictions.

The WHO uses terms such as ‘disorder’ and ‘addiction’ to describe the risks of game participation. However, there is a growing body of research highlighting the positive effects and benefits of game participation. Recent studies have explored the utility of gaming by examining cognitive functions rooted in brain cognitive function, such as motor control skills and executive functions, which are manifested through the hand coordination and visual information in the brain [7,8]. Specific research has delved into differences in attention and memory between expert and non-participant groups based on levels of gaming participation [9], cognitive function differences based on game genres [10], and the use of games for therapeutic purposes in children with attention deficit hyperactivity disorder (ADHD). Furthermore, there have been studies examining cognitive function improvement in the elderly through game participation [11,12]. Given the wide range of research participants and purposes, it is evident that research on game participation encompasses a diverse array of subjects and purposes. Recent studies have shown that games have a positive impact on cognitive function not only in the elderly but also in younger age groups, including children and adolescents [7,13]. However, most of the previous studies have primarily employed single-variable measurements or cross-sectional approaches, leading to limitations in interpretation. Thus, there is a need for multifaceted observations through various measurements to fully understand the effects of game participation.

Brain cognitive function comprises various sub-factors, such as attention, memory, spatial perception, executive function, and motor control skills [14]. Relying solely on a single-measurement variable in previous research has limitations in explaining overall cognitive functions. To address this limitation, tools for assessing intelligence, cognitive abilities, and similar constructs have been developed over the years. Intelligence assessments using Raven Progressive Matrices [15] provide a measurement tool that can exclude the influence of sociocultural differences. Various versions of this tool, adapted for different age groups and conditions, have been developed and utilized. Additionally, the Stroop test [16], a longstanding selective attention assessment tool, continues to be employed in medical and educational fields, with modern computer program versions available. In this study, in addition to the aforementioned assessments, cognitive function assessment tools such as the Tower of London and the Corsi block test were used to elucidate the impact of game participation on cognitive functions.

Furthermore, previous research found a relationship between game participation and the activation of the central nervous system through sensory neurons for visual perception and immediate motor neurons for rapid motor responses. Game participation has been shown to enhance motor skill learning [17], and differences have been observed in game-related tasks that involve actual manipulation of devices like the mouse, depending on the game participation [18]. The rapid decision-making and response requirements during gaming suggest that changes in neural plasticity can be anticipated through game participation. Therefore, this study not only aims to assess reaction times but also to evaluate performance in a real gaming environment as an indicator of motor control skills.

Therefore, the aim of this study is to highlight the positive aspects of gaming participation. To achieve this goal, this study explored the impact of adolescents’ levels of gaming participation on cognitive functions and motor control skills. Utilizing a variety of measurements targeting adolescents engaged in game participation, this study distinguishes itself from previous research, which primarily focused on single-variable approaches. Furthermore, it contributes valuable foundational data to the body of research explaining the positive changes and benefits of game participation.

The research hypotheses of this study are as follows:

First, there will be differences between groups in cognitive function and motor control skills depending on the frequency of game participation.

Second, there will be differences between groups in cognitive function and motor control skills depending on the play time of game participation.

Third, there will be differences between groups in cognitive function and motor control skills depending on school classification (middle and high school).

## 2. Methods

### 2.1. Participants

This study was conducted using a purposive sampling method targeting adolescents who regularly participate in video games. We recruited 64 middle school students and 66 high school students who agreed to undergo measurements to evaluate their cognitive function and motor control skills according to their participation in games. A total of 130 students were evaluated in this experimental study (mean of age = 14.78, 99 males and 31 females). To control for errors stemming from differences in measurement methods, particularly tasks that require responses using a mouse and keyboard, this study limited the participants to those who use their right hand to operate the mouse during gaming participation. Additionally, individuals who could not use both eyes due to eye diseases, surgery, or other reasons that may affect visual focus and eye movement characteristics during the task performance were excluded from participant recruitment.

### 2.2. Procedure and Research Ethics

The participants first responded to a brief questionnaire about their personal information and game participation. They provided responses regarding the number of days per week they engaged in game participation, the amount of time they spent gaming, and the primary games they would play. After the questionnaire and a brief explanation about the measurement program, they proceeded with the actual measurements. Participants undertook a test program consisting of 10 cognitive function and motor control skill tasks. Participants engaged in the measurements using various methods such as computer mouse, keyboard, pen, and verbal responses, depending on the measurement content. The measurements were conducted on Saturday mornings when students did not have regular classes for 5 months.

This study was approved by the Institutional Review Board (IRB) of Seoul National University, Korea (approval number: 2302/003-011). Research administration tasks required for a study targeting adolescents were prepared, including obtaining consent from guardians and school principals. Based on IRB approval, the recruitment of participants and obtaining parental consent were conducted using home communication letters and school bulletin boards.

### 2.3. Measurement

#### 2.3.1. Trail Making Test (TMT)

TMT is a symbol linking task in which participants connect ‘numbers’ and ‘numbers + letters’ sequentially with lines. It is a complex test that requires visual perception, visual scanning, motor speed, compound visual injections (visual scanning), and agility. In this study, we utilized the TMT-KL (Korean Letter) version included in the Korean version of the CERAD diagnostic assessment (CERAD-K) by Woo et al. [19] for measurement. Before conducting the measurements, participants were instructed to perform the task as quickly as possible without lifting the pen from the paper. First, the participants completed TMT-A using only ‘numbers (1~25)’. After TMT-A, the participants conducted TMT-B, which consisted of ‘numbers (1~13) + Korean letters’. Since all the participants in the study were Korean, we used a version that included Korean characters. A brief practice page was provided before each task, and each measurement point was marked with ‘start’ and ‘end’ indicators to make it easy to recognize task completion. The time (in seconds) taken to complete each task was collected as data.

#### 2.3.2. Corsi Block Test (CBT)

This test measures non-verbal short-term working memory capacity, as introduced by Corsi [20]. In practice, it involves using blocks for assessment. However, in this study, data was collected using an online measurement method (PsyToolKit.org, accessed on 1 January 2023) via PC internet access. The measurement method involves both remembering the sequence in which a total of nine blocks displayed on the monitor in random colors blink and responding with a mouse. The difficulty increases as the number of blinking blocks increases, starting with two blocks and going up to nine blocks. Two trials are provided for each stage, and if the participant does not succeed within the given attempts, the measurement is terminated. The data collected includes the maximum number of blocks that the participants successfully remembered in order. It was designed for participants to press the ‘Done’ button after completing the input.

#### 2.3.3. Tower of London (TOL)

This assessment was originally developed by Shallice [21] in the field of neuropsychology and is a prominent test used to evaluate cognitive executive functions related to planning (problem-solving, working memory, cognitive flexibility, etc.). Originally, it involved physically moving beads by hand, but in this study, we conducted measurements in an online environment using the TOL Inquisit program developed for PC use (Millisecond, WA, USA). The measurement method involves moving placed beads to match the arrangement of beads on presented mission cards, with a limited number of moves allowed for each problem. A total of 12 problems were provided, and for each problem, the participants had three attempts. Scores were calculated based on a system where if a participant successfully solved a problem within one attempt, they received 3 points, and for each additional attempt used, 1 point was deducted. The difficulty of the items gradually increased with factors like the number of allowed moves and changes in stick length. Data collection involved recording the scores obtained by the participants for solving the 12 problems.

#### 2.3.4. Stroop Test

This task, developed by Stroop [16], involves responding to colors in three different conditions: ‘single-color words’, ‘color-congruent’, and ‘color-incongruent.’ This task is a prominent measure of selective attention and was utilized in the assessment using a version created with the PsychoPy Builder program (Open Science Tools Ltd., Nottingham, England and Wales). The measurement method involves reading aloud the words and colors presented in three different conditions in three different environments, with a total of 12 ‘words’ displayed on the screens of different conditions. Data were collected by recording the time (in seconds) it took for participants to verbally read in each condition. The Stroop task is based on the interference principle, where automatic information processing, which involves analyzing the shape of words and thinking about their meanings and names, progresses faster than conscious information processing, which involves analyzing colors and naming their names [14]. In this study, the Stroop test was selected as a measure of a cognitive function factor, with the aim of not differentiating between the general population and individuals with attention disorders but periodically examining non-participants in gaming or in improvements related to gaming participation. The time taken to complete the task was measured manually using a stopwatch, and a simplified version of the task was used with four rows and three columns.

#### 2.3.5. Raven Progressive Matrices (RPM)

The measurement task in this study aimed to measure deductive ability, which is one of the general intelligence factors proposed by Spearman [22], using the well-known intelligence test RPM developed by Raven [7]. Unlike other intelligence tests, RPM assesses deductive ability by requiring participants to identify the rules governing the arrangement of shapes and deduce the missing shape in a pattern. This task is relatively free from the influence of sociocultural backgrounds, such as learning environments and levels. Furthermore, since intelligence and attention are considered important determinants of academic achievement [23,24], this task was chosen as a measure of cognitive factors in the context of examining their relationships with other variables among adolescents. Prior research, such as Liu et al.’s study [25] had already introduced improvement in the deductive abilities of children engaged in regular gaming through RPM test results. The standard RPM version (Raven SPM) consists of five sets (A to E) with varying difficulty levels, comprising a total of 60 items with 12 questions per set. However, for the purpose of assessing attention concentration rather than sustained attention, only seven items of a medium difficulty from a 3 × 3 array were used in this study. PsychoPy Builder software was used in this study, and participants were asked to input their answers via keyboard after viewing the provided arrays. Each item had to be answered within 70 s, and the number of correct answers was collected as data.

#### 2.3.6. Multi-Tasking

The measurement task in this study was developed based on research conducted by the Game Science Institute in Korea [26]. It assesses the immediate recognition of the match between the presented cards in terms of ‘letter-number’ and ‘border color.’ Participants used both hands to respond, with left-hand fingers pressing designated response keys (A, S keys) to indicate whether the letter and number matched and right-hand fingers using keys on the keyboard (K, L keys) to respond to the border color. Similar to the TMT, Korean characters were used for number matching to account for the characteristics of the study participants. Before the measurement, participants received sufficient explanations and practiced utilizing the items. The actual measurement was conducted only after participants completed the practice items, and if there were any difficulties in understanding, additional explanations were provided to ensure comprehension. To account for participants’ understanding and measurement errors, the measurement was repeated twice, with a total of 10 items, and the number of correct answers in participants’ responses was collected as data.

#### 2.3.7. Key-Mapping

Similar to the Multi-tasking task mentioned above, this measurement was also conducted based on the Game Science Institute’s research [26]. It is designed to assess participants’ ability to adapt and cope with randomly set directional keys on a keyboard. The measurement method involves completing a specified path using the W (↑), A (←), S (↓), and D (→) keys, which are commonly used as directional keys in FPS (first-person shooter) games. In the first trial, participants perform the task with default directional key settings. In the second to fourth measurement trials, participants perform the task with randomly set directional keys (note that the directional keys are set the same way in the second to fourth trials). This task, developed to examine cognitive functions such as attention, memory, and spatial perception manifested through repeated measurements under randomly set conditions. The time taken for each trial was collected as data.

#### 2.3.8. Grid Shot

Grid Shot is from the online program AimLab, which is available on the STEAM gaming platform and is based on the FPS game genre. There might be a significant relationship between participating in specific computer games and motor control skills [12]. AimLab offers various practice tasks, and among them, the Grid Shot task involves accurately and quickly hitting multiple randomly generated targets. The measurement duration was set to 15 s, and to account for errors or variability, the task was repeated twice. After every completion of the task, a summary score, along with accuracy, the number of generated targets, and the total number of successfully hit targets within 15 s was displayed on the monitor screen. Data were collected through screenshots. Also, the measurement was controlled by setting mouse sensitivity and using the right-hand mouse operation method.

#### 2.3.9. Sphere Track

This measurement task also utilized another practice task from the game AimLab, as mentioned earlier. The content of this task involves tracking a single target that randomly moves within the screen using a focal cursor, focusing on assessing accurate and rapid tracking abilities. Similar to the Grid Shot task, the measurement duration was set to 15 s, and to account for errors or variability, the task was repeated twice. After every completion of the task, the average target tracking time, error bias, accuracy in target tracking compared to time, and total score after completing the task were displayed. Data were collected through screen captures. Control measures for this task were the same as for the Grid Shot task, and particular emphasis was placed on providing sufficient guidance before the measurement to prevent unnecessary actions, such as habitual mouse clicks, during the assessment.

#### 2.3.10. Reaction Time

This task is a representative assessment of motor control skills [27], focusing on how quickly individuals react to changes in colors displayed on the screen (from red to green) by perceiving them and activating the motor nerves of the central nervous system through the sensory nervous system. The measurement was created using the PsychoPy Builder program and participants reacted by clicking the mouse. Data were collected in units of 1/1000 s, and data were gathered by averaging the results of 10 repeated measurements. To control participants’ responses through prediction, the time at which the color changed on the screen during each of the 10 repeated measurements was set to be accepted arbitrarily. In this study, data were collected by conducting two sets of 10 repeated measurements (Table 1). 

### 2.4. Statistical Analysis

Data analysis was performed using the IBM SPSS statistics program (ver. 21.0), with a statistical significance level set at 0.5. First, descriptive statistics were shown to examine the distribution and normality of measurement values for each variable. Cognitive function and motor control skills were validated for normality through Levene’s homogeneity of variance test. To verify the differences between groups according to participation level and participation time, a one-way analysis of variance (ANOVA) and Scheffe’s post hoc test were conducted, and when the equal variance assumption was rejected, Welch’s test was used. Also, an independent variable *t*-test was conducted to determine the differences between the two groups according to school classification. In addition, the effect size of statistically significant group differences was calculated through Cohen’s *f* value in the case of analysis of variance and was interpreted through Cohen’s *d* value in the case of the *t* test. The level of statistical significance was set at 0.5.

## 3. Results

### 3.1. Test Performance According to the Frequency of Game Participation

To examine the effect of the frequency of game participation on cognitive function and motor control skills between groups (4 groups: every day, 5~6 days, 3~4 days, 1~2 days), one-way ANOVA was performed. The test performance of each group according to the frequency of game participation is shown in Table 2 below. If the result of Levene’s homogeneity of variance test did not have equal variance, Welch’s test was used to examine the differences between groups.

The result of this study of research participants who regularly participated in video games indicated that there were no differences between groups according to the frequency of game participation in every cognitive function and motor control skill test.

### 3.2. Test Performance According to the Play Time of Game Participation

The group difference of cognitive function and motor control skills according to the play time of game participation are shown in Table 3 below. To examine the effect of the play time of game participation on the test performance between groups (4 groups: over 3 h, 2~3 h, 1~2 h, under 1 h), one-way ANOVA was performed. The results of this study of research participants who regularly participated in games showed statistically significant differences between groups according to the time of game participation in several cognitive function and motor control skill tests.

Significant differences between groups were found in tests that mainly reflected the ability to operate the mouse and keyboard. First, the results of the ANOVA were as follows: ‘KEY_CON (*F* = 2.858, *p* = 0.40)’, ‘GRID1_SCORE (*F* = 5.610, *p* = 0.01)’, ‘GRID2_SCORE (*F* = 4.175, *p* = 0.07)’, ‘SPHERE1_SCORE (*F* = 3.343, *p* = 0.21)’, ‘SPHERER1_ACCURACY (*F* = 3.579, *p* = 0.16)’, ‘SPHERE2_SCORE (*F* = 3.814, *p* = 0.12)’, and ‘SPHERER1_ACCURACY (*F* = 3.829, *p* = 0.12)’.

In addition, the effect size of the play time of game participation on cognitive function and motor control skill test performance was confirmed through Cohen’s *f* values, and as shown in Table 3, every effect size of the play time of game participation were confirmed as medium (Cohen’s *f* > 0.25) [28].

### 3.3. Test Performance According to School Classification

To verify the effects of school classification (middle school and high school) on cognitive function and motor control skills between the groups, an independent *t*-test was performed. As a result, it was found that there was a difference between groups only in the ‘MULTI_1 (*p* = 0.00)’ and ‘MULTI_2 (*p* = 0.05)’ performance, and there was no difference between groups in other test performances.

As shown in Table 4, the effect size of the school classification on ‘MULTI_1’ and ‘MULTI_2’ were confirmed through Cohen’s *d* value. According to Cohen’s theorem [26], the effect size of ‘MULTI_1’ was large (Cohen’s *d* = 0.830), and the effect size of ‘MULTI_2’ was medium (Cohen’s *d* = 0.501). Additionally, most test performances showed similar mean values, and there were no statistically significant group differences except for the Multi-tasking test.

## 4. Discussion

This study aimed to examine the relationship between game participation among adolescents and their cognitive function and motor control skills, applying a variety of measurement tasks. This experimental study is a follow-up study of a previous study that confirmed the difference between game participation and non-participation groups and is significant as basic data for understanding the relationship to health according to the level of game participation by targeting 130 adolescents who regularly participated in games. Discussions during the interpretation process, based on the results of the test performance according to the level of game participation, are as follows.

Firstly, there were no statistically significant differences between groups in cognitive function and motor control skill performance depending on the frequency of game participation. In our previous study [18], which revealed differences between participating and non-participating groups targeting general adults, there were differences in cognitive function and motor control skill test performance depending on the frequency of game participation. However, there was no significant difference in this study of adolescents who regularly participated in games. Accordingly, the results of this study, which showed that there was no effect of frequency on research participants who were already regularly participating in games, require more consideration in interpreting the results than simply comparing participation in the control group. This means that frequency does not play a significant role in the development of cognitive functions and learning of manipulative abilities through games. In addition, as explained in previous studies [29,30] that suggest short-term application effects, the results of this study also support the findings that cognitive function and motor control ability can be activated through short-term stimulation (game participation). These results suggest that, in fact, the frequency of game participation does not significantly affect the improvement of adolescents’ cognitive function and motor control skills.

Secondly, differences in cognitive function and motor control skills according to the play time of game participation showed statistically significant differences between groups in some test performances. This study stands out for its distinctiveness from previous research by not depending on single-measurement variables but instead examining changes in cognitive function through various measurement tasks related to game participation. In addition to measurement tasks that have already been verified through previous research in the medical and educational research fields, this study was able to verify the discriminatory power in tasks developed by researchers. Since the tests that showed differences between groups were mainly conducted under conditions similar to a video game environment, using arrow keys and a mouse, it can be seen that they reflected manipulation skills learned through game participation. These results suggest that participation time in gaming needs to be considered important in order to master manipulative skills. In addition, our results support previous studies [6,9,12] that reported that game participation has a positive effect on cognitive function. In particular, even within the sample that regularly participates in games, it is noteworthy that there are differences in task performance depending on the level of game participation time. Additionally, group differences were observed in both the score and accuracy of all trials in the Sphere Track test, which can be interpreted as reflecting proficiency in mouse manipulation and executive functions related to visual perception information. Although this study did not consider types of game participation, it can be interpreted that the ability to perceive visual information and tracking performance using a mouse was effectively learned through game participation. These results provide evidence that motor learning can occur through game participation, similar to the rapid response to stimuli perceived in a similar context in the brain.

Thirdly, in this study, in order to examine whether the influence of grade (period of learning participation) was involved in previous analysis of other results, differences between groups according to school classification were confirmed. As a result, it was found that there was only a difference between groups in the Multi-tasking1 and Multi-tasking2 performance, and there were no differences between groups in other task measurements. Specifically, the Multi-tasking performance is a task used to measure the level of executive function that requires the coordination of attention, memory, and motor control skills because it requires responding to matching letters, numbers, and colors with both hands. Therefore, it can be interpreted that as in general intelligence tests, the influence of the participant’s age and learning level, sociocultural background [31], language ability, etc. [32] may have had an effect. Nevertheless, in certain task performances such as the Stroop test, middle school students showed high average values; this study also has limitations in that it did not take into account game participation according to school classification, so there is a need for attention to this in the interpretation of the results. Accordingly, in follow-up research, it is necessary to examine the effects of games on cognitive function and motor control skills by considering the level of game participation along with grade classification.

Lastly, this study has several limitations in interpreting the results. Firstly, the study did not differentiate participants’ gaming device types (PC, mobile, console, etc.) and their level of participation (frequency and playtime) based on the gaming device. This limitation restricts the explanation of which type of participation is effective in influencing cognitive functions and motor control skill test performances. The tests primarily utilized a mouse and keyboard, and differences in proficiency with these devices could have had an impact. For this reason, future research should consider the influence of gaming device types and genres. Secondly, while this study considered school classification for sample size, it did not account for the participants’ gender. Since gender differences could influence the level of game participation and cognitive and motor development, there are limitations in interpreting differences based on the school level. Therefore, it is deemed necessary to include gender considerations in future research. Additionally, this study did not consider the time when the research participants began participating in games regularly. Therefore, there are limitations in interpretation as group differences caused by the current frequency of game participation and the level of mastery were not controlled.

## 5. Conclusions

This study revealed that regular game participation among adolescents can have positive effects and changes on cognitive function and motor control skills. Some research has classified behaviors related to game participation under terms like disease or gaming disorders, but there is still insufficient evidence for the negative aspects of game participation. By contrast, there are studies that report positive effects and changes associated with game participation. Notably, this study distinguishes itself from previous research that relied on single-measurement tests by utilizing a range of cognitive function and motor control skill tests. Furthermore, applying these measurements to over 100 adolescents engaged in regular game participation allows substantial support to be provided for the interpretative process. As a result, the findings of this study can serve as foundational data for explaining the positive relationship between game participation and the health of adolescents. It suggests that rather than viewing game participation as inherently risky, it highlights the importance of self-regulation in daily life. Lastly, given the positive effects of game participation on attention, memory, executive function, and motor control skills demonstrated in this study, the need to regard game participation as a behavior that can contribute to productivity rather than simply discouraging it is implied.

## Figures and Tables

**Table 1:** Summary of the test program.

Type	Test	Response Tool	Unit Of Data
Cognitive Function	Trail Making Test	Pen	Time Taken (s)
	Corsi Block Test	Mouse	Score
	Tower of London	Mouse	Score
	Stroop Test	Read aloud	Time Taken (s)
	Raven Progressive Matrices	Keyboard	Score
	Multi-tasking	Keyboard	Score
	Key-mapping	Keyboard	Time Taken (s)
Motor Control Skill	Grid Shot	Mouse	Score
	Sphere Track	Mouse	Score
	Reaction Time	Mouse	Time Taken (s)

**Table 2:** Test performance according to frequency of game participation.

Test	Frequency (n = 17, 27, 50, 36)	Mean (SD)	*F*	*p*
TMT_A	1. Every day	18.82 (4.16)	0.591	0.622
	2. 5~6 days	20.48 (4.63)		
	3. 3~4 days	20.81 (6.04)		
	4. 1~2 days	20.34 (5.34)		
TMT_B	1. Every day	49.40 (15.22)	0.882	0.452
	2. 5~6 days	56.98 (17.06)		
	3. 3~4 days	53.77 (19.92)		
	4. 1~2 days	57.83 (22.06)		
CBT	1. Every day	6.11 (0.99)	1.642	0.183
	2. 5~6 days	6.11 (1.08)		
	3. 3~4 days	6.28 (1.24)		
	4. 1~2 days	5.69 (1.36)		
TOL	1. Every day	30.94 (4.78)	0.449	0.718
	2. 5~6 days	32.07 (2.64)		
	3. 3~4 days	31.56 (3.39)		
	4. 1~2 days	31.94 (3.57)		
STROOP_NEU	1. Every day	4.99 (0.70)	0.890	0.449
	2. 5~6 days	5.11 (1.15)		
	3. 3~4 days	5.15 (0.99)		
	4. 1~2 days	5.49 (1.72)		
STROOP_CON^W^	1. Every day	5.91 (1.10)	2.434	0.076
	2. 5~6 days	6.36 (1.49)		
	3. 3~4 days	5.75 (0.99)		
	4. 1~2 days	6.56 (1.72)		
STROOP_INC	1. Every day	7.30 (1.20)	0.149	0.930
	2. 5~6 days	7.56 (1.73)		
	3. 3~4 days	7.49 (1.63)		
	4. 1~2 days	7.63 (2.07)		
RPM^W^	1. Every day	3.41 (1.97)	0.778	0.508
	2. 5~6 days	2.92 (1.68)		
	3. 3~4 days	2.98 (1.30)		
	4. 1~2 days	2.75 (1.27)		
MULTI_1	1. Every day	5.70 (3.01)	0.678	0.567
	2. 5~6 days	6.18 (3.10)		
	3. 3~4 days	6.26 (2.83)		
	4. 1~2 days	5.41 (2.91)		
MULTI_2^W^	1. Every day	7.70 (2.05)	1.804	0.150
	2. 5~6 days	6.51 (2.80)		
	3. 3~4 days	7.42 (2.12)		
	4. 1~2 days	6.58 (2.35)		
KEY_CON	1. Every day	15.65 (2.82)	1.177	0.321
	2. 5~6 days	17.54 (6.84)		
	3. 3~4 days	17.21 (4.12)		
	4. 1~2 days	18.33 (5.01)		
KEY_RAN1	1. Every day	36.71 (7.39)	2.258	0.085
	2. 5~6 days	43.16 (11.13)		
	3. 3~4 days	44.38 (11.06)		
	4. 1~2 days	42.81 (10.68)		
KEY_RAN2	1. Every day	29.81 (5.58)	1.796	0.151
	2. 5~6 days	33.53 (9.30)		
	3. 3~4 days	35.22 (8.95)		
	4. 1~2 days	32.74 (8.72)		
KEY_RAN3	1. Every day	27.38 (6.74)	1.035	0.380
	2. 5~6 days	31.00 (8.97)		
	3. 3~4 days	30.54 (8.40)		
	4. 1~2 days	28.74 (7.66)		
GRID1_Score	1. Every day	9087.41 (1804.98)	2.131	0.100
	2. 5~6 days	8715.00 (2319.34)		
	3. 3~4 days	8390.92 (1927.45)		
	4. 1~2 days	7783.08 (1743.47)		
GRID1_Accuracy	1. Every day	87.30 (8.63)	1.015	0.389
	2. 5~6 days	90.47 (6.18)		
	3. 3~4 days	88.13 (7.80)		
	4. 1~2 days	87.51 (7.08)		
GRID2_Score	1. Every day	10,202.47 (2029.14)	2.172	0.095
	2. 5~6 days	9600.00 (2646.22)		
	3. 3~4 days	9184.12 (2060.06)		
	4. 1~2 days	8685.11 (1942.70)		
GRID2_Accuracy^W^	1. Every day	91.27 (7.17)	0.273	0.845
	2. 5~6 days	92.61 (5.16)		
	3. 3~4 days	91.34 (5.35)		
	4. 1~2 days	91.34 (8.20)		
SPHERE1_Score	1. Every day	18,664.70 (4470.60)	2.466	0.065
	2. 5~6 days	15,764.77 (5313.65)		
	3. 3~4 days	15,242.00 (6013.55)		
	4. 1~2 days	14,203.50 (5838.47)		
SPHERE1_Accuracy	1. Every day	66.18 (10.26)	1.806	0.149
	2. 5~6 days	59.08 (13.15)		
	3. 3~4 days	57.28 (15.02)		
	4. 1~2 days	56.62 (16.95)		
SPHERE2_Score	1. Every day	19,369.11 (5580.76)	1.921	0.130
	2. 5~6 days	15,720.92 (5940.74)		
	3. 3~4 days	16,399.40 (5617.46)		
	4. 1~2 days	15,561.88 (5766.30)		
SPHERE2_Accuracy	1. Every day	67.58 (13.02)	1.891	0.134
	2. 5~6 days	58.83 (14.30)		
	3. 3~4 days	60.59 (13.42)		
	4. 1~2 days	58.69 (13.44)		
REACTION1^W^	1. Every day	271.84 (26.32)	1.000	0.395
	2. 5~6 days	271.67 (26.75)		
	3. 3~4 days	263.06 (24.51)		
	4. 1~2 days	273.21 (39.80)		
REACTION2	1. Every day	258.68 (22.42)	0.173	0.915
	2. 5~6 days	263.22 (26.19)		
	3. 3~4 days	261.52 (32.76)		
	4. 1~2 days	258.54 (28.64)		

Note: Test^W^ marks indicate that Welch’s test was performed.

**Table 3:** Test performance according to the play time of game participation.

Test	Play Time (n= 18, 35, 59, 18)	Mean (SD)	*F*	*p*	Cohen’s *f*	Post Hoc
TMT_A	1. Over 3 h	19.64 (5.17)	1.217	0.306	-	-
	2. 2~3 h	20.59 (5.48)				
	3. 1~2 h	19.78 (5.24)				
	4. Under 1 h	22.37 (5.48)				
TMT_B	1. Over 3 h	53.33 (15.01)	0.278	0.841	-	-
	2. 2~3 h	53.55 (14.84)				
	3. 1~2 h	55.36 (22.67)				
	4. Under 1 h	58.24 (20.72)				
CBT	1. Over 3 h	5.83 (1.15)	1.998	0.118	-	-
	2. 2~3 h	5.97 (1.12)				
	3. 1~2 h	6.32 (1.20)				
	4. Under 1 h	5.61 (1.46)				
TOL	1. Over 3 h	31.55 (3.14)	0.846	0.471	-	-
	2. 2~3 h	30.97 (3.51)				
	3. 1~2 h	31.94 (3.62)				
	4. Under 1 h	32.38 (3.39)				
STROOP_NEU	1. Over 3 h	5.23 (1.19)	0.575	0.633	-	-
	2. 2~3 h	5.26 (0.92)				
	3. 1~2 h	5.09 (1.08)				
	4. Under 1 h	5.52 (2.11)				
STROOP_CON	1. Over 3 h	6.13 (1.10)	0.220	0.882	-	-
	2. 2~3 h	6.09 (1.21)				
	3. 1~2 h	6.21 (1.68)				
	4. Under 1 h	5.89 (1.47)				
STROOP_INC	1. Over 3 h	6.97 (1.25)	1.139	0.336	-	-
	2. 2~3 h	7.78 (1.68)				
	3. 1~2 h	7.63 (1.88)				
	4. Under 1 h	7.22 (1.61)				
RPM	1. Over 3 h	2.77 (1.62)	0.285	0.836	-	-
	2. 2~3 h	2.85 (1.47)				
	3. 1~2 h	3.08 (1.45)				
	4. Under 1 h	2.94 (1.47)				
MULTI_1	1. Over 3 h	5.61 (2.47)	1.067	0.366	-	-
	2. 2~3 h	5.34 (2.84)				
	3. 1~2 h	6.40 (3.07)				
	4. Under 1 h	5.88 (2.94)				
MULTI_2	1. Over 3 h	6.22 (2.31)	2.403	0.071	-	-
	2. 2~3 h	7.11 (2.21)				
	3. 1~2 h	7.50 (2.26)				
	4. Under 1 h	6.16 (2.70)				
KEY_CON	1. Over 3 h	15.13 (3.01)	2.858	0.040 *	0.260	1 < 4 *
	2. 2~3 h	17.81 (5.73)				
	3. 1~2 h	17.11 (4.65)				
	4. Under 1 h	19.72 (4.96)				
KEY_RAN1	1. Over 3 h	38.73 (7.34)	1.270	0.288	-	-
	2. 2~3 h	44.05 (9.36)				
	3. 1~2 h	42.44 (12.73)				
	4. Under 1 h	44.86 (8.06)				
KEY_RAN2	1. Over 3 h	30.83 (7.70)	0.719	0.543	-	-
	2. 2~3 h	34.13 (8.36)				
	3. 1~2 h	33.56 (9.79)				
	4. Under 1 h	34.58 (6.02)				
KEY_RAN3	1. Over 3 h	28.48 (7.12)	0.620	0.603	-	-
	2. 2~3 h	29.95 (8.00)				
	3. 1~2 h	29.30 (8.96)				
	4. Under 1 h	31.89 (6.52)				
GRID1_Score	1. Over 3 h	9420.44 (1809.48)	5.610	0.001 **	0.366	1 > 4 **, 3 > 4 *
	2. 2~3 h	8231.11 (1898.38)				
	3. 1~2 h	8588.44 (1981.76)				
	4. Under 1 h	6952.94 (1520.97)				
GRID1_Accuracy	1. Over 3 h	87.64 (9.30)	0.722	0.541	-	-
	2. 2~3 h	88.14 (7.53)				
	3. 1~2 h	89.23 (6.40)				
	4. Under 1 h	86.47 (8.37)				
GRID2_Score	1. Over 3 h	10,220.22 (1949.19)	4.175	0.007 **	0.314	1 > 4 *, 3 > 4 *
	2. 2~3 h	9176.88 (1994.75)				
	3. 1~2 h	9463.66 (2307.19)				
	4. Under 1 h	7833.38 (1779.89)				
GRID2_Accuracy	1. Over 3 h	90.10 (7.66)	1.057	0.370	-	-
	2. 2~3 h	90.54 (6.55)				
	3. 1~2 h	92.31 (6.20)				
	4. Under 1 h	92.78 (6.17)				
SPHERE1_Score	1. Over 3 h	17,310.38 (4633.88)	3.343	0.021 *	0.283	1 > 4 *
	2. 2~3 h	16,432.71 (5609.06)				
	3. 1~2 h	15,494.61 (5779.83)				
	4. Under 1 h	11,970.05 (5784.82)				
SPHERE1_Accuracy	1. Over 3 h	63.02 (10.97)	3.579	0.016 *	0.293	1 > 4 *, 2 > 4 *
	2. 2~3 h	61.49 (13.71)				
	3. 1~2 h	58.74 (15.39)				
	4. Under 1 h	49.19 (15.42)				
SPHERE2_Score	1. Over 3 h	18,918.50 (6301.08)	3.814	0.012 *	0.301	1 > 4 *
	2. 2~3 h	17,046.94 (5440.06)				
	3. 1~2 h	16,367.11 (5621.71)				
	4. Under 1 h	12,839.00 (4958.61)				
SPHERE2_Accuracy	1. Over 3 h	66.34 (14.80)	3.829	0.012 *	0.303	1 > 4 *
	2. 2~3 h	62.14 (12.97)				
	3. 1~2 h	60.60 (13.15)				
	4. Under 1 h	51.95 (12.65)				
REACTION1	1. Over 3 h	275.93 (23.64)	1.627	0.186	-	-
	2. 2~3 h	267.05 (32.11)				
	3. 1~2 h	264.32 (30.17)				
	4. Under 1 h	279.78 (30.29)				
REACTION2	1. Over 3 h	274.37 (24.16)	2.088	0.105	-	-
	2. 2~3 h	255.20 (21.33)				
	3. 1~2 h	258.38 (32.28)				
	4. Under 1 h	265.15 (31.32)				

Note: * *p* < 0.5, ** *p* < 0.1, Scheffe’s post hoc test was performed.

**Table 4:** Test performance according to school classification.

Test	Mean (SD)		*t*	*p*	Cohen’s *d*
	High School (n = 66)	Middle School (n = 64)			
TMT_A	20.34 (5.50)	20.34 (5.23)	−0.003	0.998	-
TMT_B	51.77 (15.08)	55.32 (22.75)	−1.928	0.056	-
CBT	6.02 (1.43)	6.11 (0.99)	−0.437	0.663	-
TOL	31.68 (3.80)	31.70 (3.19)	−0.035	0.972	-
STROOP_NEU	5.29 (1.35)	5.14 (1.13)	0.711	0.478	-
STROOP_CON	6.04 (1.48)	6.22 (1.44)	−0.734	0.464	-
STROOP_INC	7.75 (1.87)	7.29 (1.55)	1.506	0.135	-
RPM	2.79 (1.35)	3.14 (1.58)	−1.367	0.174	-
MULTI_1	7.05 (2.61)	4.80 (2.81)	4.732	0.000 ***	0.830
MULTI_2	7.61 (2.01)	6.45 (2.56)	2.850	0.005 **	0.501
KEY_CON	17.13 (4.65)	17.66 (5.24)	−0.618	0.538	-
KEY_RAN1	43.55 (11.31)	41.82 (10.11)	0.920	0.359	-
KEY_RAN2	33.58 (8.58)	33.38 (8.86)	0.133	0.894	-
KEY_RAN3	29.31 (7.55)	30.15 (8.75)	−0.586	0.559	-
GRID_Score	8437.56 (2137.75)	8322.64 (1814.03)	0.330	0.742	-
GRID_Accuracy	88.64 (7.19)	88.03 (7.69)	0.464	0.644	-
GRID2_Score	9473.17 (2293.70)	9051.30 (2072.64)	1.099	0.274	-
GRID2_Accuracy	91.00 (7.09)	91.39 (5.91)	0.364	0.717	-
SPHERE1_Score	15,978.98 (5910.41)	15,027.53 (5570.42)	0.944	0.347	-
SPHERE1_Accuracy	59.83 (14.98)	57.64 (14.77)	0.840	0.402	-
SPHERE2_Score	17,265.91 (5902.62)	15,537.31 (5531.34)	1.722	0.088	-
SPHERE2_Accuracy	62.62 (13.73)	58.55 (13.47)	1.705	0.091	-
REACTION1	269.91 (32.38)	267.67 (27.89)	0.421	0.674	-
REACTION2	257.65 (27.15)	263.81 (30.48)	−1.217	0.226	-

Note: ** *p* < 0.1, *** *p* < 0.01.

## Data Availability

The data are available upon request from the corresponding author.
